# Uncovering Low-Level Maternal Gonosomal Mosaicism in X-Linked Agammaglobulinemia: Implications for Genetic Counseling

**DOI:** 10.3389/fimmu.2020.00046

**Published:** 2020-02-12

**Authors:** Jacques G. Rivière, Clara Franco-Jarava, Mónica Martínez-Gallo, Aina Aguiló-Cucurull, Laura Blasco-Pérez, Ida Paramonov, María Antolín, Andrea Martín-Nalda, Pere Soler-Palacín, Roger Colobran

**Affiliations:** ^1^Pediatric Infectious Diseases and Immunodeficiencies Unit, Vall d'Hebron Research Institute, Hospital Universitari Vall d'Hebron, Universitat Autònoma de Barcelona, Barcelona, Spain; ^2^Jeffrey Model Foundation Excellence Center, Barcelona, Spain; ^3^Immunology Division, Department of Cell Biology, Physiology and Immunology, Vall d'Hebron Research Institute, Hospital Universitari Vall d'Hebron, Autonomous University of Barcelona, Barcelona, Spain; ^4^Department of Clinical and Molecular Genetics, Hospital Universitari Vall d'Hebron, Barcelona, Spain

**Keywords:** X-linked agammaglobulinemia (XLA), Bruton agammaglobulinemia, gonosomal mosaicism, genetic counseling, BTK mutation

## Abstract

X-linked agammaglobulinemia (XLA) is a clinically and genetically well-defined immunodeficiency and the most common form of agammaglobulinemia. It is characterized by susceptibility to recurrent bacterial infections, profound hypogammaglobulinemia, and few or no circulating B cells. XLA is caused by mutations in the *BTK* gene, which encodes Bruton's tyrosine kinase (BTK). Because of its X-linked recessive inheritance pattern, XLA virtually only affects males, and the mother is the carrier of the mutation in 80–85% of the males with this condition. In the remaining 15–20% of the cases, the affected male is considered to have a *de novo* mutation. Here, we present the case of a child with a diagnosis of XLA caused by a missense mutation in the *BTK* gene (c.494G>A/p.C165Y). Apparently, his mother was wild type for this gene, which implied that the mutation was *de novo*, but careful analysis of Sanger electropherograms and the use of high-coverage massive parallel sequencing revealed low-level maternal gonosomal mosaicism. The mutation was detected in various samples from the mother (blood, urine, buccal swab, and vaginal swab) at a low frequency of 2–5%, and the status of the patient's mutation changed from *de novo* to inherited. This study underscores the importance of accurately establishing the parents' status on detection of an apparently *de novo* mutation in a patient, as inadvertent low-level mosaicism may lead to misinterpretation of the risk of recurrence, vital for genetic counseling.

## Introduction

X-linked agammaglobulinemia (XLA, OMIM #300755) is a prototypical primary immunodeficiency (PID), which was first described in 1952 by the pediatrician Ogden Carr Bruton (1). XLA is characterized by increased susceptibility to bacterial infections, a huge reduction in all serum immunoglobulin isotypes, and a profound decrease or absence of peripheral blood B lymphocytes (typically <2%) (2, 3). The genetic defect underlying XLA was not discovered until 1992, when two independent groups found that it was caused by mutations in the *BTK* gene, which encodes Bruton's tyrosine kinase (BTK) (4, 5). BTK is involved in signal transduction from the B-cell immunoglobulin receptor, and BTK deficiency results in a profound block of B-cell differentiation in bone marrow at early stages (6). Several studies (including patients from different countries and ethnic groups) coincide in that more than 85% of males with presumed XLA have mutations in *BTK* (3, 7–10). Although it is difficult to accurately define the incidence of XLA, it likely varies from 1/100,000 to 1/250,000 live births.

The *BTK* gene, located on the long arm of the X chromosome (Xq22.1), contains 19 exons and codes for a 659-amino acid protein. Since the gene was discovered, hundreds of mutations were reported, and most of them were compiled in the BTKbase: http://structure.bmc.lu.se/idbase/BTKbase/ (11). The mutations are scattered almost uniformly throughout the gene and include a variety ranging from point mutations to large gene rearrangements. Several studies focused on the molecular analysis of large cohorts of XLA patients. Collectively, they report that the most common mutations are missense, followed by frameshift, splicing, and non-sense mutations, whereas gross deletions and mutations affecting the promoter are the least frequent (3, 8, 9, 12–15). The genotype–phenotype association in XLA was addressed by several groups with discrepant results, but overall, a strong correlation has not been established (3, 12, 16–18). Interestingly, although XLA is considered a fully penetrant disorder, hypomorphic variants presenting with a leaky phenotype (i.e., low, but not absent, immunoglobulin levels and decreased, but not absent, B cells) also exist and may account for up to 10% of the patients (19–23).

Because of its X-linked recessive inheritance pattern, XLA almost exclusively affects males, with some extremely rare exceptions (24), and in about 80–85% of the affected males, the mother is the carrier of the mutation (8, 25). In the remaining 15–20% of the cases, the affected male is considered to carry a *de novo* mutation, unless he has a sibling with the same mutation. In this latter situation, maternal mosaicism is strongly suspected, as the mother is an obligate carrier. However, even in families with a sporadic patient carrying a *de novo* mutation (i.e., the mother apparently does not have the mutation), maternal mosaicism cannot be excluded. In current common practice, a mutation is considered *de novo* if direct sequencing by the Sanger method detects the mutation in the patient's peripheral blood DNA, but not in that of either of the parents. However, Sanger sequencing cannot detect mutants with low allele fractions (typically <10% of mutated alleles). Therefore, it is conceivable that parental mosaicism may be underdetected, especially when the allele fraction is low (26). The use of next-generation sequencing (NGS) technologies can greatly facilitate detection of mosaicism, especially low-level mosaicism (27), and this is important to unambiguously classify a mutation as inherited or *de novo*.

Here, we report on a child diagnosed with XLA caused by a missense mutation in the *BTK* gene (c.494G>A/p.C165Y). His mother was apparently wild type for this gene, but careful analysis of the Sanger sequencing results and the use of massive parallel sequencing revealed low-level, maternal gonosomal mosaicism. Although the frequency of the mutation was low (<5.5%) in all tissues analyzed, transmission to the patient indicated that it was present in germ cells, a fact with important implications for genetic counseling.

## Methods

### Patients and Samples

Peripheral blood was obtained from the proband, and peripheral blood, urine, and buccal and vaginal swabs were obtained from the mother.

Written informed consent for the studies reported here and for the publication of this case report was obtained from the patient's legal representatives, and a separate consent was given by the mother for her samples, in accordance with the procedures of the ethics review board of Hospital Universitari Vall d'Hebron [code: PR(AG)69/2016].

### Sanger Sequencing

All exons and flanking regions of the *BTK* gene were sequenced in the patient, whereas only the exon containing the mutation was sequenced in the patient's mother. Genomic DNA was extracted from EDTA-containing whole-blood samples using the QIAamp DNA blood mini kit (Qiagen; Hilden, Germany) according to the manufacturer's instructions. A polymerase chain reaction (PCR) technique was carried out to amplify the 19 exons of *BTK* and their flanking regions (primers and PCR conditions are available upon request), and purified PCR products were sequenced on an ABI 3100 DNA sequencer using the BigDye Terminator sequencing kit 3.1 (Applied Biosystems; Foster, VA, USA).

### Targeted Gene Sequencing

Samples from the patient's mother were analyzed to establish the percentage of mosaicism in different specimens (peripheral blood, urine, and buccal and vaginal swabs). PCR products containing the exon of interest were used as DNA input to generate libraries, which were sequenced with the MiSeq platform (Illumina, San Diego, CA).

DNA was quantified with a Qubit 2.0 Fluorometer (Thermo Fisher Scientific, MA, USA), and 300–900 ng of DNA was fragmented using NEBNext dsDNA Fragmentase (New England Biolabs, Ipswich, MA) to obtain fragments of ~200 bp. Then, end repair, ligation of the adapter for Illumina, and sample indexing by a small amplification were carried out using the NEBNext Ultra DNA library prep kit for Illumina and NEBNext Multiplex Oligos for Illumina Dual Index Primers Set 1 (New England Biolabs, Ipswich, MA). All necessary purifications and size selections to recover only the fragments of interest were done using AMPure XP beads (Beckman Coulter). Libraries were quality-evaluated using QIAxcel (Qiagen) and quantified with a Qubit 2.0 Fluorometer. The DNA sample libraries were then mixed in equimolecular amounts and sequenced in a MiSeq instrument (Illumina, San Diego, CA), using the 500-cycle MiSeq reagent kit v2 with a paired-end run of 2 × 251-bp reads. All procedures were performed according to the manufacturer's instructions.

### Bioinformatics Analysis

The data analysis pipeline included adapter and quality trimming of the Illumina FASTQ sequences using Trimmomatic (28), and alignment to the human genome GRCh37 (hg19) using BWA-MEM. Low-frequency variants in *BTK* were called using the Pisces variant caller (29) and annotated with ANNOVAR (30).

### Flow Cytometry Assessment of Intracellular BTK Expression

Peripheral whole blood was collected in vacutainer tubes containing ethylene-diamine-tetra-acetic acid (EDTA) as anticoagulant (BD-Plymouth, PL6 7BP, UK). A 50-ml amount of blood was incubated with a mix of specific conjugated monoclonal antibodies (CD14–PB and CD19–PCy7). Cells were then fixed and permeabilized according to the manufacturer's instructions for the PerFix-nc Kit (Beckman Coulter). After permeabilization, samples were stained with anti-BTK–PE or isotype control IgG1–PE (MiltenyiBiotec) and gently mixed for 30 min at room temperature (RT) in the dark. Samples were treated with 1 ml of lysing solution (VersaLyse, Beckman Coulter), vortexed, and incubated for 15 min at RT in the dark, and then washed with phosphate-buffered saline (PBS), stored at RT in the dark, and analyzed within 1 h. Samples were acquired with a Navios EX flow cytometer (Beckman Coulter) and analyzed with Kaluza software.

## Results

### Case Presentation

An 8-month-old boy was referred to our hospital for respiratory distress, fever, and fatigue. He had been born at term from a non-consanguineous family with no relevant prior family history. Before this episode, he had been admitted twice for bronchitis at 3 and 6 months of age. During one of these episodes, he showed severe neutropenia and received a broad-spectrum antibiotic. Neutrophil counts fully recovered a few days later without further treatment. On physical examination, there were no palpable lymph nodes or tonsillar tissue. Lung auscultation detected bilateral crackles and diminished breath sounds. The chest X-ray showed bilateral infiltrates with no pleural effusion, thus establishing the diagnosis of acute bilateral pneumonia. Complete blood count at the age of 8 months was normal. Immunoglobulin levels were very low (IgA <10 mg/dl, IgM 12 mg/dl, and IgG 135 mg/dl) (Table 1). The patient had a clear X-linked agammaglobulinemia (XLA) immunophenotype, with normal T- and NK-cell numbers, and profound B-cell deficiency (1%, 50 cells/μl). The lymphocyte proliferation assay was normal. These laboratory findings, together with the absence of lymph nodes and tonsils, suggested the diagnosis of XLA, and immunoglobulin replacement therapy was started.

**Table 1:** Immunological parameters in the study patient.

	**8 months**	**Age-matched reference values**
Leukocytes (×10^9^/L)	10.79	5.8–17.8
Neutrophils (%)	↓19.6	35–75
Neutrophils (×10^9^/L)	2.1	1.5–5
Lymphocytes (%)	↑55.1	20–50
Lymphocytes (×10^9^/L)	6	3.4–9
T cells CD3^+^ (%)	89.97	50–77
CD3^+^ (×10^9^/L)	4.56	2.4–6.9
CD4^+^ (%)	55.95	33–58
CD4^+^ (×10^9^/L)	2.84	1.4–5.1
CD8^+^ (%)	31.82	13–26
CD8^+^ (×10^9^/L)	1.61	0.6–2.2
Index	1.76	1.6–3.8
CD19^+^ (%)	↓1	13–35
CD19^+^ (×10^9^/L)	↓0.05	0.7–2.5
CD56^+^CD16^+^ (%)	5.78	2–13
CD56^+^CD16^+^ (×10^9^/L)	0.29	0.1–1.0
IgG (mg/dl)	↓135	196–1,045
IgA (mg/dl)	↓ <10	8–90
IgM (mg/dl)	↓12	40–140

### Identification of the *BTK* p.C165Y Mutation in the Patient and His Mother: Germinal Mutation vs. Gonosomal Mosaicism

**Figure 1:**
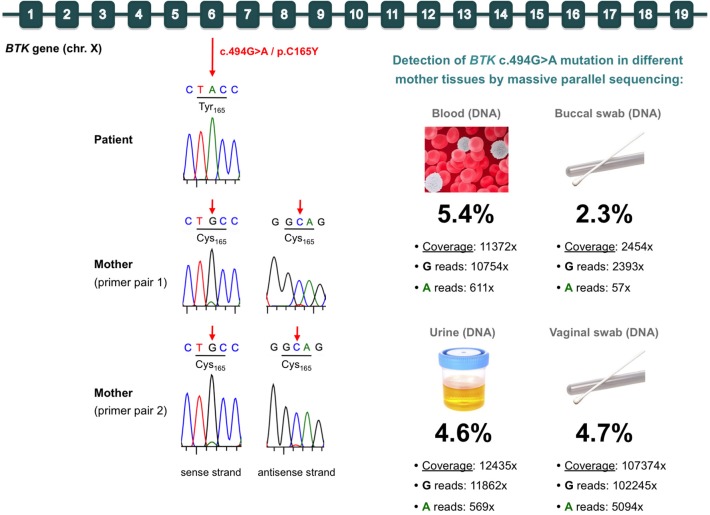
Identification of the *BTK* mutation in the patient (germinal mutation) and his mother (gonosomal mosaicism). **(Top)** Diagram of the *BTK* gene, indicating the c.494G>A/p.C165Y mutation in exon 6. **(Left)** Electropherograms of Sanger sequences from the patient and his mother. In the mother, two different primer pairs were used, which consistently showed a very small peak corresponding to the mutated allele in both the sense and antisense strands. **(Right)** Massive parallel sequencing results for detection of the mutation in various samples from the mother. The total coverage, reads containing each allele, and percentage of the mutated allele are indicated for each sample.

Based on the clear suspicion of XLA, Sanger sequencing of the *BTK* gene was performed, and the hemizygous missense mutation c.494G>A/p.C165Y was identified (Figure 1). This mutation, located in exon 6 of *BTK*, was classified as *likely pathogenic* according to the American College of Medical Genetics and Genomics rules (31). The mutation was not found in the ExAC or Gnomad database, but it had been previously reported in a family including two boys with XLA (32). Therefore, we considered *BTK* p.C165Y to be the causal mutation of XLA in our patient.

We also sequenced the mother's DNA, and she was, apparently, wild type. However, careful analysis of the Sanger chromatogram (forward sequence) revealed a very small adenine peak at the c.494 position, which would correspond to the mutated allele (Figure 1). Analysis of the reverse sequence showed a barely visible peak at c.494, which would also correspond to the mutated allele (Figure 1). To rule out selective allele amplification, we designed a second pair of primers to amplify and sequence the *BTK* region of the c.494G>A/p.C165Y mutation in the mother. The results were exactly the same, strongly suggesting the presence of maternal gonosomal mosaicism (Figure 1). To address this issue and to quantify to what extent the mutation was present in the mother, we performed targeted deep sequencing of the p.C165Y *BTK* mutation using DNA from hematological and non-hematological samples. We obtained high, on-target depth coverage (from 2,454× to 107,374×), and we detected the *BTK* p.C165Y mutation in all samples analyzed, at similar low frequencies: blood (5.4%), urine (4.6%), buccal swab (2.3%), and vaginal swab (4.7%) (Figure 1).

### Flow Cytometry Assessment of Intracellular BTK Expression

**Figure 2:**
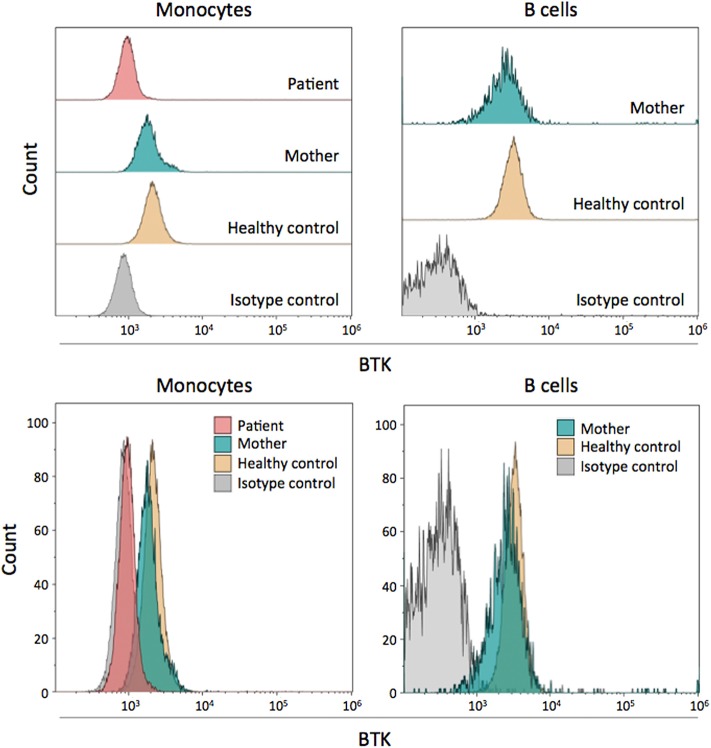
Flow cytometry assessment of intracellular BTK expression. BTK protein expression is shown in the patient, his mother, and a healthy control. Monocytes and B cells were both examined. In the patient, only monocytes were analyzed due to the lack of B cells. The results are shown individually and grouped for easy comparison.

BTK protein expression in monocytes and B cells of the patient and his mother was analyzed by flow cytometry. The patient's monocytes showed an absence of BTK expression, confirming that the p.C165Y mutation led to complete BTK deficiency (Figure 2). BTK expression in the patients' mother was within the normal range in both monocytes and B cells, indicating that her low-level mosaicism did not have a significant impact and could not be reliably detected by flow cytometry (Figure 2). It is difficult to establish whether the slight decrease in BTK expression observed in the mother was caused by her mosaicism, as it was not consistently observed using other healthy controls (data not shown). In fact, her low-level mosaicism (~5%) is below the interindividual variation of BTK expression in healthy controls, and this may mask its effect.

## Discussion

Mosaicism is a type of post-zygotic variation, in which two or more genotypically distinct cell lines derived from a single zygote are present in an individual. Although mosaicism is increasingly recognized as a source of genetic variation, it is still an underestimated genetic phenomenon responsible for the development of human phenotypes in health and disease (33). Depending on the developmental stage in which a mutation occurs, mosaicism can be classified into one of three categories: (1) germline mosaicism (also known as gonadal mosaicism) refers to genetic variation emerging in cells that develop into gonads (ovaries and testicles). It is one cause of *de novo* variation in the next generation; (2) somatic mosaicism, affecting cells other than germline cells; and (3) gonosomal mosaicism, a combination of germline and somatic mosaicism that refers to mosaic variants present in both somatic and germline lineages (34, 35).

In relation to PID, mosaicism was recently reported as an unexpectedly frequent phenomenon in patients with autoinflammatory disorders (36–39). An example of this is the striking estimate that 0.5–19% of patients with cryopyrin-associated periodic syndromes (CAPS) show mosaicism (40). In PIDs other than autoinflammatory disorders, mosaicism was found to a lesser extent. Perhaps the most widely known and classic example was provided in 2004 by Holzelova et al., who described a group of patients with autoimmune lymphoproliferative syndrome (ALPS) showing somatic mosaicism and heterozygous *FAS* mutations in polyclonal double-negative T cells (41). Later, other groups confirmed and extended this finding, reporting that somatic mutations account for up to 15% of ALPS cases (42). Only very recently has the role of gene mosaicism in PID patients been extensively investigated. In a study led by JI Arostegui including 92 families with a single member carrying a presumably *de novo* mutation, six families (6.5%) were identified in which one parent carried the mutation in the form of gonosomal mosaicism (43). These results also support the notion that this phenomenon is remarkably frequent in patients with autoinflammatory diseases (43).

In XLA, around 15–20% of the patients were considered to have *de novo* mutations because the mother was not a carrier, as typically determined by the Sanger method. However, a presumed *de novo* mutation may, in fact, be inherited by undetected mosaicism in a parent who lacks symptoms. Since the discovery of *BTK* as the genetic defect underlying XLA (4, 5), several studies pointed out that parental mosaicism had to be present in some cases where a parent was expected to be an obligate carrier (44–47). For example, in 2001, Sakamoto et al. described two XLA brothers with the same BTK mutation, born of a mother with no evidence of the mutation. The authors Sanger sequenced the *BTK* gene in the blood samples and buccal mucosal cells from the mother, and no mutations were detected. The fact that a non-carrier mother had two affected sons with the same mutation led the authors to suggest maternal gonadal mosaicism (44). A recent study by Segundo et al. reported a case of XLA in which the patient and his daughter carried the same *BTK* mutation, but the mother had no mutations in *BTK*, findings that strongly suggest maternal gonadal (or gonosomal) mosaicism. In this case, the authors only sequenced *BTK* from blood DNA (Sanger method) and did not investigate mosaicism in the mother (45). To date, the only study that has fully demonstrated parental mosaicism in XLA is the aforementioned report by JI Arostegui, in which BTK gonosomal mosaicism was found in two unrelated families with XLA patients (43). These authors demonstrated mosaicism in DNA from the mothers' whole blood samples using NGS, but they did not investigate DNA from other tissues.

To our knowledge, the present study is the first to comprehensively report low-level maternal gonosomal mosaicism in XLA with evidence from DNA analysis of various tissues. We found that the low-level mosaicism detected in the mother's blood (5.4% of NGS reads) was consistently present at a similar frequency in the other samples analyzed (urine, and buccal and vaginal swab). Detection and quantification of mosaicism is important for establishing the diagnosis of disease, assessing the risk of recurrence, and guiding genetic counseling. NGS is a proven, reliable technique to identify low-level mosaicism, which may go undetected by conventional Sanger sequencing (27). This is crucial when addressing the parent's status for a previously identified mutation because the presence of inadvertent low-level mosaicism may lead to misinterpretation of a genetic testing result. In particular, an inherited case may be misinterpreted as *de novo* due to low-level mosaicism in a carrier parent. In addition, estimation of the risk of recurrence can be highly inaccurate when mosaicism is not considered during genetic counseling (48). In the mothers of XLA patients with germline *BTK* mutations, the probability of recurrence would be ~50%, whereas in true *de novo* mutations, the risk would be the same as in the general population (negligible in terms of genetic counseling). In XLA patients with a mosaic parent, it can be assumed that the recurrence risk would be dependent on the percentage of germ cell progenitors that harbor the mutation (49). In malesDNA analysis in sperm can be informative for risk assessment, but in a woman, the level of mosaicism in gonads cannot be easily determined (50). In our patient's mother, the mutation was detected at a similar low frequency (≤ 5.5%) in all tissues analyzed, which represented the three germ layers (ectoderm, endoderm, and mesoderm). Although it is tempting to speculate that the results would be similar in gonads, this assumption cannot be made, as the level of mosaicism varies substantially between different tissues and body locations, even within the same embryonic lineage (50). In the light of these considerations, a precise estimation of recurrence risk is difficult in the case reported here. At present, a conservative approach has been followed, offering by default the same risk as in germline *BTK* mutations (up to 50%).

## Data Availability Statement

The raw data supporting the conclusions of this article will be made available by the authors, without undue reservation, to any qualified researcher.

## Ethics Statement

Written informed consent for the studies reported here and for the publication of this case report was obtained from the patient's legal representatives, and a separate consent was given by the mother for her samples, in accordance with the procedures of the Ethics Review Board of Hospital Universitari Vall d'Hebron [code: PR(AG)69/2016].

## Author Contributions

JR was the principal clinician in charge of the patient's care. He collected clinical data and wrote part of the manuscript. CF-J participated in the study design and collected laboratory data. MM-G performed the flow cytometry analysis. AA-C provided technical support for sample collection and the Sanger sequencing process. LB-P, IP, and MA performed the massive parallel sequencing and data analysis. AM-N and PS-P were clinicians in charge of patient care and management. RC was responsible for designing the study, performing the genetic analysis, writing the manuscript, and approving the final draft. All authors reviewed the manuscript and contributed to the final draft.

### Conflict of Interest

The authors declare that the research was conducted in the absence of any commercial or financial relationships that could be construed as a potential conflict of interest.
